# OVA12 promotes tumor growth by regulating p53 expression in human cancer cells

**DOI:** 10.18632/oncotarget.17501

**Published:** 2017-04-28

**Authors:** Renfeng Zhang, Xicai Wu, Xiangfeng Xia, Asma Khanniche, Feifei Song, Bingchang Zhang, Ying Wang, Hailiang Ge

**Affiliations:** ^1^ Department of Laboratory Medicine, Shandong Provincial Hospital Affiliated to Shandong University, Jinan, China; ^2^ Clinical Laboratory, People's Hospital of Rizhao, Rizhao, China; ^3^ Department of Radiology, The Third People's Hospital of Rizhao, Rizhao, China; ^4^ Shanghai Institute of Immunology, Shanghai Jiao Tong University School of Medicine, Shanghai, China; ^5^ Department of Pathology, Tenth People's Hospital of Tongji University, Shanghai, China

**Keywords:** *OVA12*, tumor growth, p53

## Abstract

Ovarian cancer-associated antigen 12 (*OVA12*) was first identified in an ovarian carcinoma complementary DNA (cDNA) expression library and has been shown to play an important role in tumor growth. Here, we found that overexpression of OVA12 accelerated tumor growth in different tumor cells, whereas OVA12 depletion was associated with the opposite effect. Moreover, knocking down OVA12 led to a significant increase in the protein levels of p53, and the overexpression of OVA12 significantly decreased endogenous p53 levels. In addition, OVA12 stimulated p53 polyubiquitination and degradation by the proteasome and promoted tumor growth at least partially through the p53 pathway. Taken together, these results indicate that OVA12 is a negative regulator of p53 and that inhibition of OVA12 expression might serve as a therapeutic target to restore tumor suppression.

## INTRODUCTION

Identification of appropriate tumor antigens is the first and most important step in the successful development of antigen-specific immunotherapy. Cancer-testis antigens (CTAgs) are expressed in a broad range of human tumors, but their normal expression is restricted to immunologically “privileged” tissues, including developing germ cells and trophoblastic tissues in the placenta [1]. To date, more than 100 CTAgs have been reported in the literature [2]. The widespread expression of CTAgs in tumors further supports their oncogenic activity, suggesting that they might serve as potential targets for tumor diagnosis, antigen-specific vaccination and antigen-directed immunotherapy. Thus, the search for novel CTAgs has been a continuous task in the field of tumor immunology.

Using a serological analysis of recombinant cDNA expression libraries (SEREX), which is based on immunoscreening a cDNA library derived from an ovarian cancer patient, a novel gene named *OVA12* (protein molecular weight 12 kDa) was identified [3, 4]. The gene *OVA12* is located on human chromosome 9q34.3 with an ORF of 345 bp and encodes a 114-amino-acid protein. In our previous study (ref), we showed that this gene is highly expressed in tumors and that it accelerates *in vitro* and *in vivo* tumor development. Moreover, we found that expression of OVA12 protein could resist tumor cell apoptosis induced by 5-fluorouracil through upregulation of Mcl-1 and survivin [4]. Our previous findings suggest that OVA12 is a novel CT antigen that functions as a positive regulator of tumor cell proliferation and consequently may serve as a valuable target for tumor diagnosis and therapy development. However, the molecular mechanisms of the involvement of OVA12 in the process of tumorigenesis remains unclear.

p53 is a tumor suppressor protein and a transcriptional regulator that plays an important role in cellular responses to various stress signals [5, 6]. Biochemically, p53 acts as a transcription factor that can both activate and repress gene expression [7, 8]. As such, p53 protects cells from a variety of stress signals, such as DNA damage, oncogenic insults and nucleotide depletion, by activating the transcription of a panel of genes involved in cell cycle arrest and apoptosis in addition to repressing the genes involved in antiapoptosis and cell cycle progression [9–11]. The importance of p53 as a tumor suppressor is highlighted by the fact that approximately 50% of all human cancers carry inactivating mutations in the p53 gene [12]. In certain subtypes of cancer, such as triple-negative breast cancer, mutations can be found in up to 80% of samples [13].

p53 is primarily regulated at the level of protein stability by its interacting partner, MDM2 [14, 15]. MDM2 functions as an E3 ubiquitin ligase, which mediates p53 ubiquitination and proteasomal degradation *in vivo* [16]. In addition, MDM2 binds to p53 and hinders its transcriptional activity. The MDM2 gene has been shown to be amplified and/or overexpressed in several different cancer types [17]. Surprisingly, the frequency of amplification and/or overexpression of MDM2 is relatively low in various tumors with a wild-type p53 status [18, 19]. These data suggest that other mechanisms might also regulate p53 levels.

In this study, we found that OVA12 promoted both cell proliferation *in vitro* and xenograft tumor growth *in vivo*. Moreover, loss of OVA12 resulted in a significant increase in p53 levels, and its overexpression significantly decreased endogenous p53 levels. Thus, OVA12 regulates cell proliferation and cell cycle progression in a p53-dependent manner. Collectively, these results reveal that OVA12 is a novel diagnostic and potential therapeutic target in cancer.

## RESULTS

### OVA12 promotes cell proliferation *in vitro*

**Figure 1:**
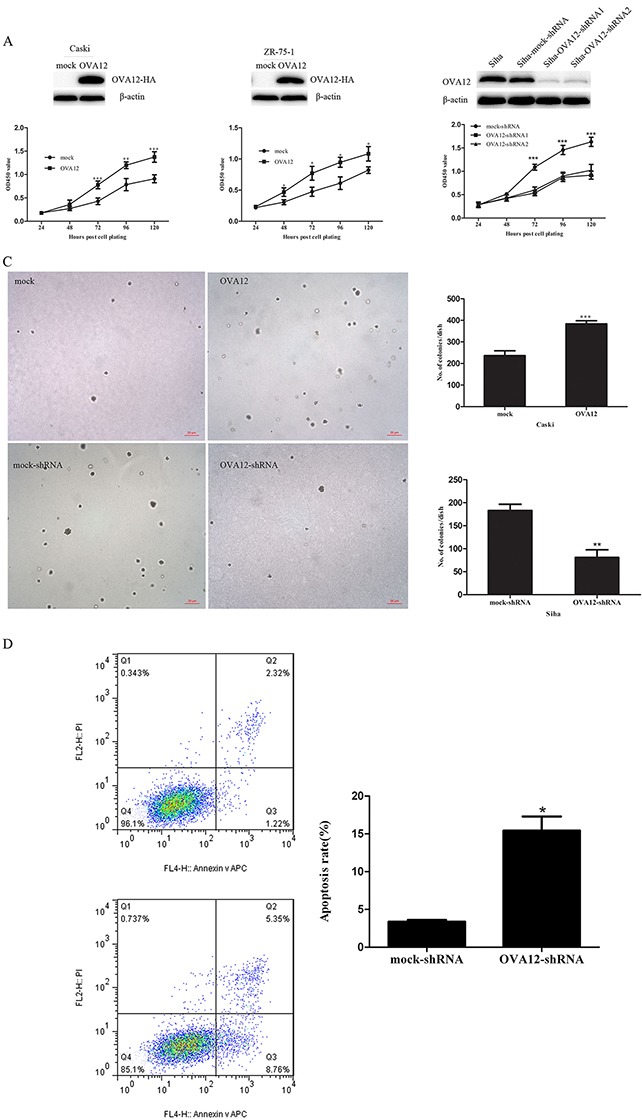
OVA12 promotes cell proliferation *in vitro* (**A**) Overexpressoin of *OVA12* enhances cell growth. Cell lines originating from the Caski and ZR-75-1 were established with stable overexpression of *OVA12* and measured by CCK-8 assay. (**B**) Depletion of *OVA12* suppresses cell growth. Cancer cells Siha were stably transfected with mock-shRNA and two *OVA12*-shRNAs and cell proliferation was examined by CCK-8 assay. (**C**) *OVA12* promotes colony formation. Soft colony formation assay was performed using *OVA12* gene-transfected Caski cells and *OVA12*-shRNA-1 transfected Siha cells. **(D)** Down regulation of OVA12 induces apoptosis. The values shown are expressed as the mean ± SD, n=3, *p<0.05 (OVA12-shRNA group vs mock-shRNA group).

The novel tumor antigen OVA12 was identified by immunoscreening a cDNA library derived from an ovarian cancer patient through a SEREX analysis. OVA12 has been shown previously to be overexpressed in diverse primary human tumor tissues and cell lines. To further determine the role of OVA12 in regulating cancer cell proliferation, we stably overexpressed OVA12 in Caski and ZR-75-1 cells. As shown in Figure 1A, cell proliferation was significantly enhanced by ectopic expression of OVA12, which is consistent with the observations of SMMC-7721 and HO8910 cells. Next, we used two shRNAs to stably knockdown OVA12 expression in Siha cells. The efficiency of OVA12 knockdown was shown in Table 1. Downregulation of OVA12 remarkably reduced the growth of Siha cells as measured by CCK-8 analysis (Figure 1B). Furthermore, a colony formation assay in soft agar showed that Caski cells stably overexpressing OVA12 produced more and larger colonies, whereas OVA12-depleted cells yielded fewer and smaller colonies than mock cells (Figure 1C). We further assessed the impact of OVA12 on cell apoptosis by FACS. As shown in Figure 1D, OVA12-shRNA transfection greatly increased apoptosis in Siha cells.

**Table 1:** The efficiency of OVA12 knockdown in tumor cells

	CT value		ΔCT	ΔΔCT
	**β-actin**	**OVA12**	**OVA12-actin**	
Siha-mock-shRNA	14.22	21.88	7.66	
Siha-OVA12-shRNA1	14.43	24.71	10.28	2.62
Siha-OVA12-shRNA2	14.33	24.16	9.83	2.17
Relative	OVA12-shRNA1/mock-shRNA			0.163
mRNA level	OVA12-shRNA2/mock-shRNA			0.222

### OVA12 promotes tumor growth *in vivo*

**Figure 2:**
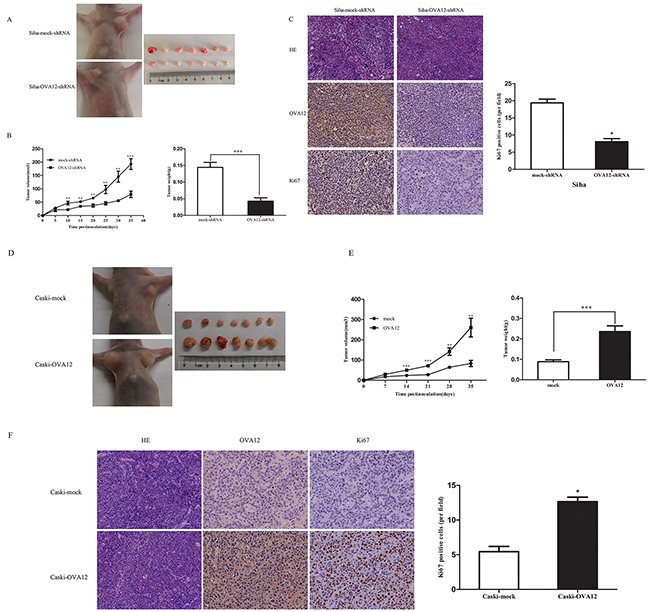
OVA12 promotes tumor growth *in vivo* (**A**) Siha-*OVA12*-shRNA or Siha-mock-shRNA cells were subcutaneously injected into the nude mice. Images are of representative mice from each injection group and all the tumors of two xenografted groups. (**B**) Tumor volumes were monitored every 5 days for 35 days and tumor weights were measured. The values shown are expressed as the Mean ± SD, n=7, **p<0.01, ***P<0.001 (OVA12-shRNA group vs mock-shRNA group). (**C**) HE staining of the tumors and immunohistological staining of the tumors using an anti-*OVA12* antibody and anti-Ki67 antibody. Xenograft tumors from Siha-*OVA12*-shRNA cells contain significantly less Ki67 positive proliferative cells. (**D**) Nude mice were injected subcutaneously with 5×10^6^cells per side for each of the indicated stable cell lines. The tumors were removed from nude mice and imaged. (**E**) Tumor growth curves and tumor weights after injection in nude mice. Tumor volumes were measured every week for 5 weeks (n=7). Tumor weights were assessed at 35 days post-injection. The values shown are expressed as the mean ± SD, n=7, **p<0.01, ***P<0.001 (OVA12 group vs mock group). (**F**) HE staining of the tumors and immunohistological staining of the tumors using an anti-*OVA12* antibody and anti-Ki67 antibody.

Because OVA12 is highly expressed in tumors and promotes cell proliferation *in vitro*, we speculated that OVA12 might enhance tumorigenesis *in vivo*. To examine this hypothesis, we performed xenograft tumor assays using Siha cells and Caski cells stably transfected with OVA12-shRNA or infected with lentivirus to induce overexpression of OVA12, respectively. The results showed that OVA12-depleted cells formed smaller tumors than control cells (Figure 2A and 2B), whereas OVA12-overexpressing cells generated larger tumors (Figure 2D and 2E). Furthermore, we observed that tumors derived from OVA12-depleted cells displayed a lower Ki67 index compared to mock cells (Figure 2C), whereas tumors originating from cells with OVA12 overexpression showed higher malignancy and much stronger proliferation ability, as indicated by HE staining and immunostaining with Ki67 antibody (Figure 2F). Taken together, these results indicate that OVA12 is a novel tumor antigen endowed with tumor promoting properties and the ability to positively regulate tumor growth.

### OVA12 negatively regulates the tumor suppressor p53

**Figure 3:**
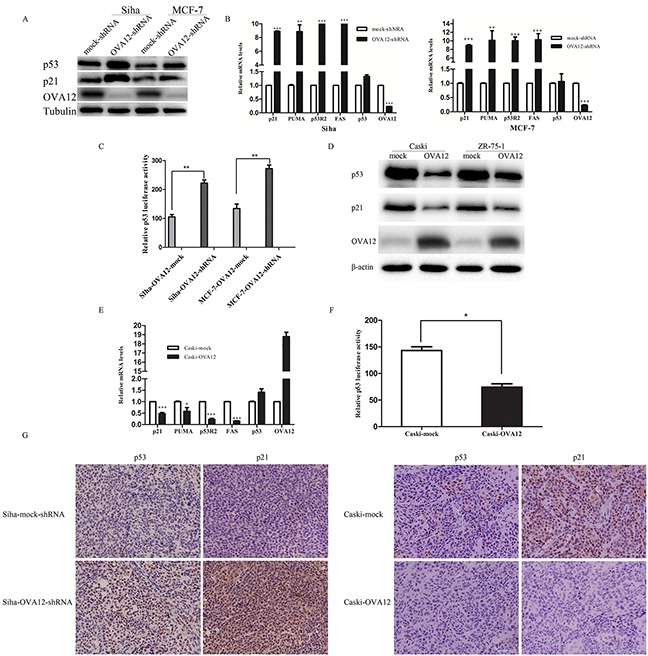
OVA12 negatively regulates p53 stability (**A**) The shRNA-mediated depletion of *OVA12* increases p53 and p21 protein levels in Siha and MCF-7 cells. (**B**) *OVA12* knockdown increases the mRNA expression of p53 downstream target genes in Siha and MCF-7 cells. (**C**) Depletion of *OVA12* promotes the luciferase activities of p53 reporter in Siha and MCF-7 cells. The values shown are expressed as the mean ± SD, **p<0.01. (**D and E**) Overexpression of *OVA12* suppresses p53 and p21 protein levels and reduces the mRNA levels of p53 downstream genes in Caski and ZR-75-1 cells. (**F**) Overexpression of *OVA12* inhibits the luciferase activities of p53 reporter in Caski cells. (**G**) IHC was performed to detect the p53 and p21 protein levels in xen-ograft tumors generated from Siha-mock-shRNA/*OVA12*-shRNA cells and Caski-mock/*OVA12* cells. Representative pictures are shown at magnifications of 400×.

To investigate the molecular mechanism by which OVA12 promoted tumor growth, we tested several signaling transduction pathways that have been previously demonstrated to be critical in tumorigenesis. We found that OVA12 knockdown resulted in an increase in p53 and p21 protein levels (Figure 3A). We further examined several p53 downstream molecules using real-time PCR and found that the mRNA levels of p21, PUMA, P53R2, and Fas were upregulated in OVA12-knockdown Siha and MCF-7 cells (Figure 3B). By contrast, p53 and p21 protein levels decreased when OVA12 was overexpressed in Caski and ZR-75-1 cells (Figure 3D). Consistently, the mRNA levels of the target genes of p53, such as p21, PUMA, P53R2, and Fas were greatly decreased in OVA12-overexpressing Caski cells (Figure 3E). Additionally, we found no change in the mRNA levels of p53 in OVA12-knockdown and OVA12-overexpressing tumor cells, suggesting a post-transcriptional regulation of p53 by OVA12. We further examined the p53 transcriptional activity using a luciferase reporter system. As shown in Figure 3C and 3F, OVA12 knockdown significantly increased the luciferase activity, whereas overexpression of OVA12 led to attenuated intensity of the p53 luciferase reporter. Consistently, we found significantly elevated expression of p53 and p21 in Siha OVA12-shRNA xenograft groups using IHC, and the reverse trend for p53 and p21 expression was observed in Caski OVA12-overexpressing xenograft groups (Figure 3G). We further examined the impact of OVA12 on mutant p53 in the MDA-MB-231 cell line carrying p53 mutation. As shown in Supplementary Figure 1A, overexpression of OVA12 did not affect the protein level of endogenous mutant p53.

### OVA12 enhances ubiquitination and degradation of p53

**Figure 4:**
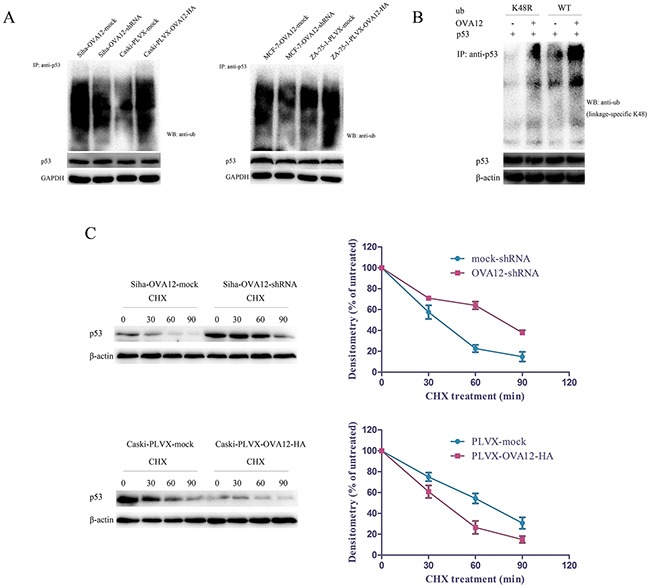
OVA12 promotes the polyubiquitination of p53 (**A**) Ubiquitination of p53 is enhanced by OVA12-overexpression while attenuated by OVA12 knockdown. (**B**) HO8910 cells were transfected with flag-OVA12, HA-UbWT, HA-UbK48R and pCMV/p53 in the indicated combinations. The cells were then treated with MG132 (10μM) for 5h. The cell lysates were subjected to immunoprecipitation followed by Western blot analysis. (**C**) Siha-mock-shRNA/OVA12-shRNA and Caski-mock/OVA12 cells were treated with CHX (25μg/ml) and harvested at indicated timepoints. The cell lysates were subjected to Western blot analysis. The data are represented as the mean ± SD of the three independent experiments.

Because we did not find any changes in p53 mRNA levels between OVA12-knockdown and OVA12-overexpressing tumor cells, we hypothesize that OVA12 promotes p53 protein polyubiquitination and proteasome-dependent degradation. In agreement with our hypothesis, the results from co-immunoprecipitation experiments showed that ectopic expression of OVA12 dramatically increased the polyubiquitination of p53 in Caski and ZR-75-1 cells (Figure 4A). By contrast, knockdown of OVA12 led to a decrease in the polyubiquitination of p53 in Siha and MCF-7 cells (Figure 4A).

Ubiquitin (Ub) chain extension can occur on a number of different lysine (K) residues on the Ub molecule, but only chain polymerization at K48 is important for substrate degradation by the proteasome [20, 21]. To examine whether OVA12 promotes K48-linked polyubiquitin chain formation of p53, we performed a ubiquitination assay with the ubiquitin mutant UbK48R, which cannot form K48-conjugated polyubiquitin chains. As shown in Figure 4B, OVA12 strongly induced the polyubiquitination of p53 in the presence of wild-type ubiquitin, but not K48R ubiquitin, suggesting that OVA12-stimulated p53 polyubiquitination occurs predominantly on the K48 of Ub. These findings reveal that OVA12 promotes tumor growth by inducing ubiquitination and degradation of p53 by the proteasome.

We further examined the half-life of p53 in the presence of the protein translation inhibitor cycloheximide (CHX) in Siha and Caski cells. When OVA12 was downregulated, the half-life of p53 was dramatically prolonged from ∼30 to ∼70 min. By contrast, ectopic expression of OVA12 noticeably shortened the half-life of p53 from ∼60 to ∼30 min (Figure 4C).

### The tumor promoting activity of OVA12 depends on p53

**Figure 5:**
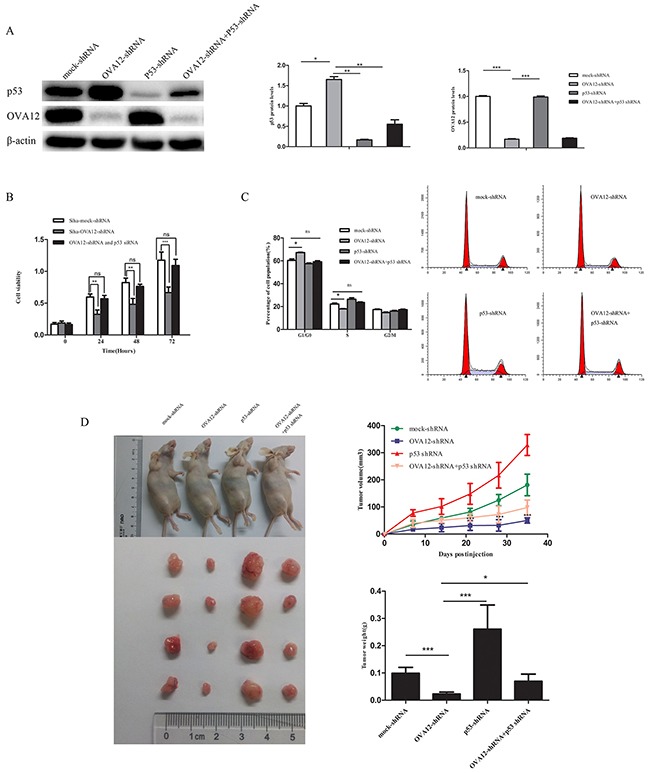
OVA12 exerts the tumor promoting activities through the p53 tumor suppressor (**A**) Siha cells were transfected with *OVA12*-shRNA and p53-shRNA alone or in combination. The cell lysates were then subjected to immunoblot analysis. (**B**) p53 knockdown attenuates the inhibitory effect on the Siha cell proliferation by *OVA12* knockdown. The cell proliferation ability was measured by CCK-8 assay. (**C**) Cell cycle analysis in Siha cells stably expressing the indicated shRNAs (n=3). (**D**) Siha cells stably expressing the indicated shRNAs were injected into nude mice. After 5-week injection, the mice were euthanized and analyzed for tumor growth. The values shown are expressed as the mean ± SD, n=4, *p<0.05, ***P<0.001.

To address the biological significance of p53 in the tumor-promoting function of OVA12, we investigated the influence of p53 knockdown on the tumorigenicity of OVA12 shRNA-transfected cells (Figure 5A). The depletion of OVA12 suppressed cancer cell proliferation, while p53 knockdown restored the cell growth rate resulting from the loss of OVA12 (Figure 5B). Cell cycle analysis using flow cytometry showed that knockdown of OVA12 caused G1 arrest in Siha cells, while silencing OVA12 and p53 together showed a marked reversal in G1 arrest (Figure 5C). Furthermore, tumor xenograft experiments also showed that the reduction of tumor volumes/weights by OVA12 downregulation was dependent on p53 function, as knocking down both OVA12 and p53 expression reversed tumor growth to the level of the control group (Figure 5D). These results strongly indicate that OVA12 promotes tumor cell growth at least partially through the p53 signaling pathway.

### OVA12 inhibits the expression of p14ARF and enhances the interaction between MDM2 and p53

**Figure 6:**
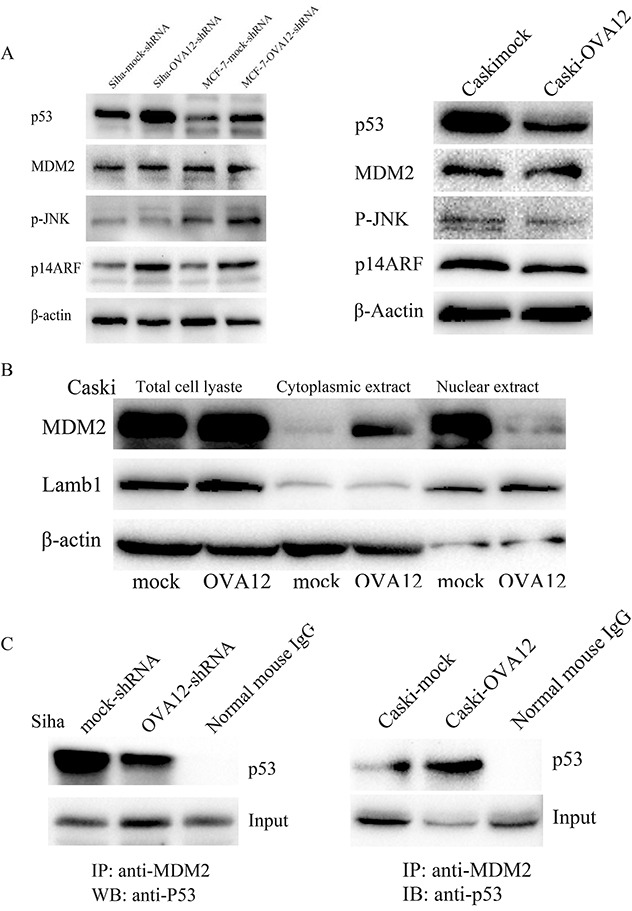
OVA12 inhibits the expression of p14ARF and enhances the interaction between MDM2 and p53 (**A**) Downregulation of OVA12 elevated the expression of p14ARF in Siha and MCF-7 cells, whereas overexpression of OVA12 inhibited the expression of p14ARF in Caski cells. (**B**) OVA12 promoted the export of MDM2 from the nucleus to the cytoplasm. (**C**) Co-immunoprecipitation analysis of the protein interaction between MDM2 and p53 showed that overexpression of OVA12 enhanced the interaction between MDM2 and p53.

To further elucidate the mechanism by which OVA12 negatively regulates the p53 pathway, we performed an immunoprecipitation (IP) experiment and found that there was no interaction between OVA12 and p53 (data not shown). Simultaneously, we investigated the protein levels of MDM2, p-JNK and p14ARF and found that downregulation of OVA12 elevated the expression of p14ARF in Siha and MCF-7 cells, whereas overexpression of OVA12 inhibited the expression of p14ARF in Caski cells (Figure 6A). p14ARF can associate with MDM2 to inhibit the ubiquitination, nuclear export and subsequent degradation of p53. Because p14ARF physically sequesters MDM2 in nucleoli, thus relieving nucleoplasmic p53 from MDM2-mediated degradation, we examined the distribution of MDM2 in the cytoplasm and nucleus in Caski cells. We observed that OVA12 promoted the export of MDM2 from the nucleus to the cytoplasm (Figure 6B). Furthermore, co-immunoprecipitation analysis of the protein interaction between MDM2 and p53 showed that overexpression of OVA12 enhanced the interaction between MDM2 and p53 (Figure 6C).

## DISCUSSION

Characterization of new tumor antigens is an active field in tumor immunology. *OVA12* was first identified from an ovarian cancer cDNA expression library through a SEREX approach. A previous study revealed that OVA12 is highly expressed in a variety of tumors and exhibits tumor-growth promoting properties in SMMC-7721 and HO8910 cells [4]. Here, we found that OVA12 also regulated tumorigenesis in other tumor cells, such as Caski, ZR-75-1 and Siha cells. Overexpression of OVA12 promoted Caski cell proliferation both *in vitro* and *in vivo*, whereas stable knockdown of OVA12 in Siha cells inhibited cell proliferation and xenograft tumor growth. Considering the critical role played by the tumor suppressor p53 in cell growth arrest and apoptosis, we investigated whether OVA12 affected p53 regulation. Interestingly, we found that knocking down OVA12 resulted in a significant increase in the p53 level and a reduction of endogenous p53 ubiquitination. Consistently, overexpression of OVA12 led to enhanced p53 ubiquitination and a decreased endogenous p53 steady-state level. In addition, OVA12 overexpression led to a significant decrease in the p53 protein half-life, whereas *OVA12* gene silencing resulted in a prolonged stability of p53.

Next, we investigated whether blocking p53 expression can rescue cell proliferation and growth arrest caused by the loss of OVA12 in Siha cells. The results showed that simultaneous inhibition of OVA12 and p53 expression by RNAi successfully rescued the OVA12-depletion phenotype, indicating that OVA12 promoted tumorigenesis through downregulation of p53. However, we did not observe a full rescue. Therefore, we speculate that p53 is only partially responsible for the tumor promoting function of OVA12 antigen. Therefore, we do not exclude other targets that OVA12 might affect in the process of tumor development.

How does OVA12 promote p53 ubiquitination? The hypothesis and molecular mechanisms underlying this process have not been previously investigated. In this study, we found that OVA12 does not physically interact with p53 (data not shown). Interestingly, we observed that OVA12 overexpression downregulated the p14^ARF^ levels, whereas knockdown of OVA12 increased the p14^ARF^ levels. The p14^ARF^ tumor suppressor is a product of the INK4a/ARF locus and is a critical regulator of p53 stability [22]. p14^ARF^ can interact with MDM2 to inhibit the ubiquitination, nuclear export and subsequent degradation of p53 [23–32]. In our study, we found that overexpression of OVA12 promoted the export of MDM2 from the nucleus to the cytoplasm in Caski cells and promoted the interaction between MDM2 and p53. Whether OVA12 promoted p53 ubiquitination through the p14^ARF^-p53 pathway is an interesting question that should be explored in the future.

## MATERIALS AND METHODS

### Cell cultures

Cell lines (Siha, MCF-7, Caski, ZR-75-1) were obtained from the Shanghai Institutes for Biological Sciences, Chinese Academy of Sciences. Siha and Caski cells were maintained in Dulbecco's modified Eagle's minimal essential medium with 10% fetal bovine serum, and MCF-7 and ZR-75-1 cells were cultured in Roswell Park Memorial Institute 1640 medium with 10% fetal bovine serum. All cell lines were cultured at 37°C in a humidified incubator with 5% CO_2_.

### Plasmid construction and establishment of OVA12-overexpressing cells

The cDNA of *OVA12* was amplified by RT-PCR using the gene-specific primers 5′-atcgGTCGACGCCACCATGTGGGCCCAGCCCTGTGCCA-3′ (Forward) and 5′- atcgGCGGCCGCTCATTAGGTTCCAGGCAGTTCAAAAACA-3′ (Reverse). The PCR products were cloned into the lentiviral expression vector pLVXpuro. Lentiviruses were produced by co-transfection of 293T cells with pLVX-mock and pLVX-OVA12 vectors and packaging vectors pSAX2 and pMD2G using Lipofectamine 2000 (Invitrogen). The viral supernatant was collected 48 h post-transfection and used to infect Caski and ZR-75-1 cells. After 48 h of infection, the infected cells were then selected with 4 μg/ml puromycin for a continuous 4-day period to generate stable transfected cell lines.

### Establishment of stable OVA12-knockdown cells

Based on the OVA12 cDNA sequence, two pairs of short hairpin RNA (shRNA) candidates were designed and introduced into a pSIREN expression vector, and they were named OVA12-shRNA1(GGAATAAAGGTACATATAGAG) and OVA12-shRNA2(GGTGTTGCTGCGTATTCCTGC). To package the virus, 293T cells were seeded in a 6-well plate 24 h prior to transfection. Either OVA12 shRNA or mock-shRNA was co-transfected with the packaging plasmid VSV-G and gag/pol using Lipofectamine 2000. At 48 h post-transfection, the viral supernatant was collected to infect target cell lines. After 48 h of infection, the infected cells were selected by exposure to 4 μg/ml puromycin for 4 days. Puromycin-resistant cells were isolated, cultured in medium containing 2 μg/ml puromycin and expanded for subsequent study.

### Cell proliferation assay

Cells (5×10^3^ per well) were plated in 96-well plates and cultured in medium with 0.5% FBS for 24 h. Then, at specific time points (24, 48, 72, and 96 h), 10 μl of CCK-8 solution was added, and the cells were incubated at 37°C for 2 h (n=6). Absorbance was measured at 450 nm using a spectrophotometer.

### Soft colony formation assay

Cells were suspended at a density of 2×10^3^ per well in medium containing 10% FBS and 0.3% low melting temperature agarose and placed on the top of solidified 0.7% agarose containing complete medium in a 6 well plate. After 2 weeks of culture, colonies were stained with 0.5% crystal violet and counted.

### Xenograft transplantation and *in vivo* tumor studies

Cells were resuspended in 100 μl of PBS and injected subcutaneously in both posterior flanks of 4-week-old female athymic nude mice (5×10^6^ cells per side). The size of the tumors was measured weekly using a caliper, and the tumor volume was calculated using the following formula: V=length×width^2^×0.5. The animals were sacrificed after 5 weeks, and the tumor bulk was harvested. All mice (Shanghai SLAC Laboratory Animal Co. Ltd., Shanghai, China) were housed in laminar flow cabinets under specific pathogen-free conditions. The study protocol was approved by the China Institutional Ethics Review Committee for Animal Experimentation.

The tumors were fixed with 4% buffered formalin, paraffin-embedded, and cut into 4-μm sections for immunohistochemical staining of OVA12, Ki67, p53 and p21 expression. Antibodies used to detect Ki67, p53 and p21 were from Santa Cruz Biotechnology; the OVA12 antibody for IHC detection was generated by our lab. Secondary antibodies against mouse or rabbit immunoglobulin G were supplied in an IHC kit from Gene Tech Company Limited (Shanghai, China).

### Western blot analysis

Cells were lysed on ice for 10 min with M-PER™ Mammalian Protein Extraction Reagent (Pierce), and the lysates were centrifuged at 14,500 rpm at 4°C for 15 min. The protein concentration of the supernatant was determined using a BCA Protein Assay Reagent Kit (Pierce). Twenty micrograms of protein was separated with 12% SDS-polyacrylamide gel electrophoresis and transferred onto a polyvinylidene difluoride membrane. Membranes were blocked for 1 h at room temperature and then incubated with primary antibodies overnight at 4°C. The membranes were then washed three times with TBST for 5 min, blotted with hoseradish peroxidase (HRP)-conjugated secondary antibody (KPL company) for 1 h at room temperature and detected with enhanced chemiluminescence reagent (MILLIPORE). The following primary antibodies were used: anti-p53 (#sc-926), anti-MDM2 (#sc-965), anti-p21 (#sc-817), anti-ubiquitin (#sc-8017) and anti-p-JNK (#sc-6254, Santa Cruz); anti-Δ-actin (Catalog #3598-100, BioVision); Anti-Ubiquitin (linkage-specific K48) antibody(ab140601, abcam). The OVA12 antibody was prepared by our lab. To prepare the OVA12 antibody, we made a polyclonal antibody against the recombinant OVA12 full-length protein. The DNA sequence corresponding to the full-length protein was subcloned into the pET-32a (+) vector. Anti-OVA12 antisera were raised in rabbits against the purified His-OVA12 protein, and further affinity-purified on a protein A column.

### Real-time PCR analysis

Total RNA was extracted from cultured cells using TRIzol reagent (Invitrogen) according to the manufacturer's instructions. First strand cDNA was synthesized using a Revert Aid First Strand cDNA synthesis kit (Fermentas). Real-time PCR was performed in a total volume of 20 μl of reaction buffer. The primers used for amplification of the cDNA were synthesized by Sangon Biotech (Shanghai, China) (Table 2). Real-time PCR was performed using a SYBR Premix Ex Taq kit in an Applied Biosystems 7500 Fast Real-Time PCR system. The real-time PCR results were analyzed using the delta CT method.

**Table 2:** Primer sequences used in real-time PCR assay

Gene	Forward	Reward
p21	TTAGCAGCGGGACAAGGAGT	CGTTAGTGCCAGGAAAGACA
PUMA	AAAGGCTGTTGTGCTGGTG	TTTGGCTCATTTGCTCTTCA
p53R2	CGGTTTGTCATCTTTCCAATC	GCTTGTTCCAGTGAGGGAGA
FAS	GGACCCTCCTACCTCTGGTT	TCCTCAATTCCAATCCCTTG
p53	GCCATCTACAAGCAGTCACAG	ATTTCCTTCCACTCGGATAAGA
OVA12	TGGGTGTTGCTGTGTATTCC	TCTTAGGTTCCAGGCAGTTCA

### Luciferase reporter assay

Siha-mock-shRNA/OVA12-shRNA, MCF-7-mock-shRNA/OVA12-shRNA, and Caski-mock/OVA12 cells were plated in 6-well plates and co-transfected with 4.0 μg of the p53 reporter plasmid pp53-TA luc and 0.4 μg of pRL-TK Renilla luciferase construct (Promega) as an internal control using Lipofectamine 2000. After a 48-h incubation, cell lysates were harvested in 500 μl of Passive Lysis Buffer, and the p53 activity was determined with a luciferase assay system (Promega) according to the manufacturer's instructions. All experiments were performed in triplicate.

### Immunoprecipitation and ubiquitination assays

Cells were treated with 10 μM MG132 for 5 h before collection and then lysed in a lysis buffer (20 mM Tris pH 7.5, 150 mM NaCl, 1 mM EDTA,1 mM EGTA, 1% Triton X-100) supplemented with protease and phosphatase inhibitors (Thermo). Cell lysates were incubated with 2 μg of p53 primary antibody overnight at 4°C. After incubation, 20 μl of protein A/G agarose beads (Thermo) were added to the cell lysates, and the mixture was incubated at 4°C on a rotating platform for 3 h. Then, the immunoprecipitates were collected by centrifugation at 1000 × g for 5 min, and the supernatant was discarded. The beads were then washed 3 times with lysis buffer and dissolved in 100 μl of 2× loading buffer for Western blot analysis. The antibodies used in the co-immunoprecipitation experiments were anti-p53 and anti-ubiquitinantibodies.

### Protein half-life detection

Siha-mock-shRNA/OVA12-shRNA and Caski-mock/OVA12 cells were plated in 6-well plates and treated with 25 μg/ml cycloheximide (CHX) at the indicated time points. Cells were lysed in M-PER™ Mammalian Protein Extraction Reagent containing protease and phosphatase inhibitors after CHX treatment. Cell lysates were then collected for Western blotting to determine the p53 protein levels.

### Cell cycle assay

Cells were trypsinized, rinsed twice with ice-cold PBS and fixed in 70% ice-cold ethanol. Then, the cells were collected by centrifugation and resuspended in 0.2 mg/ml propidium iodide containing 0.1% Triton X-100 and 1 mg/ml RNase A. The cell suspension was incubated in the dark for 30 min at 37°C and subsequently analyzed for DNA content using fluorescence-activated cell sorting (FACS) on a FACSCalibur flow cytometer (Becton Dickinson).

### Cell apoptosis assay

Cells were plated in 6-well plates 24 h before induction of apoptosis. After treatment with cisplatin for 48 h, the cells were harvested and double stained with annexin V-FITC and propidium iodide using an Annexin V-FITC Apoptosis Detection Kit (Becton Dickinson). Then, they were subjected to flow cytometric analysis and analyzed with the CELLQUEST software system. Cells in the right quadrant represented apoptotic cells.

### Statistical analysis

All the obtained data are expressed as the means±standard deviation (SD). Statistical differences were evaluated with a two-tailed Student's t-test. P values <0.05 were considered statistically significant. All statistical tests were performed using GraphPad Prism software version 5.01.

## SUPPLEMENTARY FIGURE


