# Supplementary material for: OVA12 promotes tumor growth by regulating p53 expression in human cancer cells
Source: Oncotarget. 2017 Apr 28;8(32):52854–65. doi: 10.18632/oncotarget.17501 (PMC5581076; doi:10.18632/oncotarget.17501)
Supplement: Supplementary file 1 [file oncotarget-08-52854-s001.pdf]

OVA12 promotes tumor growth by regulating p53 expression in human cancer cells

SUPPLEMENTARY MATERIALS

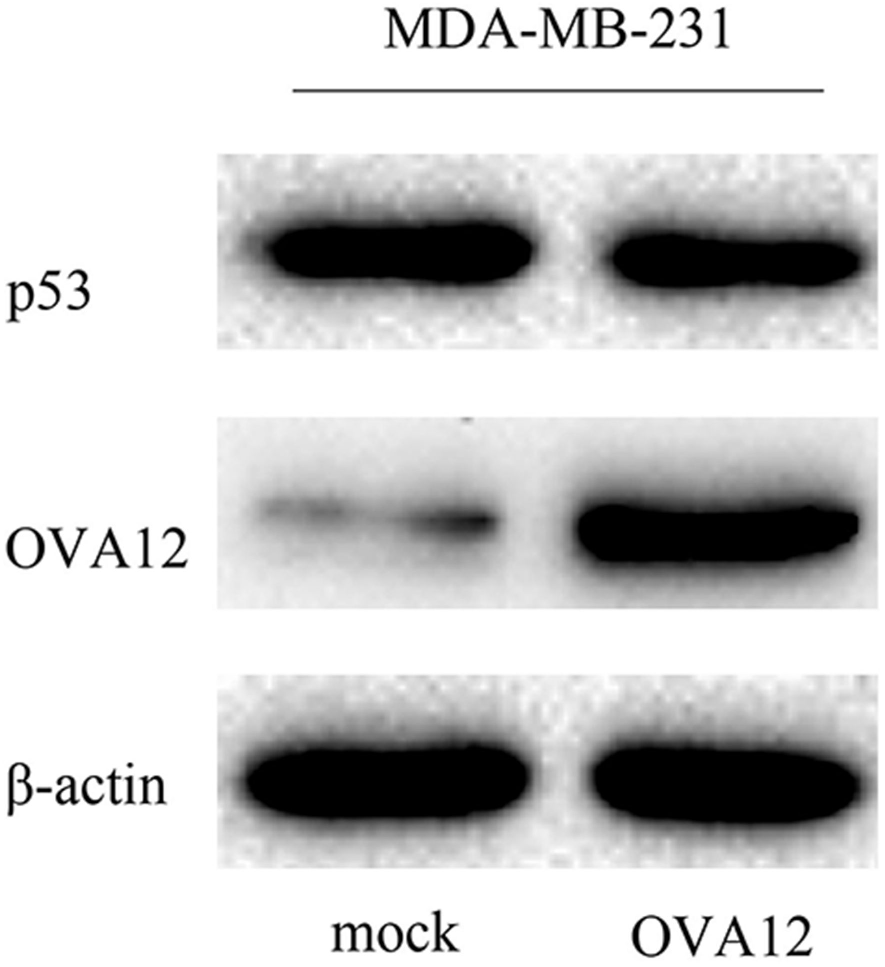

Supplementary Figure 1: Overexpression of *OVA12* did not affect the endogenous protein level of mutant p53 in MDA-MB-231 cells. MDA-MB-231 cells were transfected with pLVX-mock and pLVX-OVA12 vectors and analyzed by Western blot.
